# Supplementary material for: MetaRibo-Seq measures translation in microbiomes
Source: Nat Commun. 2020 Jun 29;11:3268. doi: 10.1038/s41467-020-17081-z (PMC7324362; doi:10.1038/s41467-020-17081-z)
Supplement: Supplementary file 10 — Supplementary Data 7 [file 41467_2020_17081_MOESM10_ESM.zip › File2/Confidence_High_Taxonomy/387001_out.krona.html]

Javascript must be enabled to view this page.

members
magnitude
magnitudeUnassigned
count
unassigned
taxon
rank

387001\_out

20

superkingdom
2
20

1239
20
phylum

20
186801
class

order
186802
20

20
541000
family

genus
20
216851

10

SRS012849\_contig\_number\_24961SRS016954\_contig\_number\_12196SRS018623\_contig\_number\_11585SRS019685\_contig\_number\_contig-100\_25913.45891SRS057480\_contig\_number\_contig-100\_39970.39971SRS098061\_contig\_number\_12048SRS098881\_contig\_number\_701SRS143372\_contig\_number\_7814SRS144537\_contig\_number\_contig-100\_4153.270649SRS893373\_contig\_number\_1636
853
species

species
1971605
10

SRS015663\_contig\_number\_30158SRS018817\_contig\_number\_17426SRS019030\_contig\_number\_contig-100\_7213.7213SRS048870\_contig\_number\_5962SRS050998\_contig\_number\_contig-100\_3756.83535SRS065504\_contig\_number\_25625SRS075878\_contig\_number\_contig-100\_11845.50865SRS142923\_contig\_number\_contig-100\_34252.34253SRS143417\_contig\_number\_contig-100\_582.137668SRS149325\_contig\_number\_15532
